# Fat mass index as a screening tool for the assessment of non-alcoholic fatty liver disease

**DOI:** 10.1038/s41598-022-23729-1

**Published:** 2022-11-23

**Authors:** Shengkui Zhang, Lihua Wang, Miao Yu, Weijun Guan, Juxiang Yuan

**Affiliations:** 1 Department of Epidemiology and Health Statistics, School of Public Health, North China University of Science and Technology, 21 Bohai Road, Caofeidian Xincheng, Tangshan, 063210 Hebei China; 2 Department of Epidemiology and Health Statistics, School of Basic Medicine, Institute of Basic Medical Sciences, Chinese Academy of Medical Sciences, Peking Union Medical College, Beijing, 100730 China

**Keywords:** Gastrointestinal diseases, Metabolic disorders

## Abstract

Non-alcoholic fatty liver disease (NAFLD) is replacing hepatitis B as the leading cause of chronic liver disease in China. The purpose of this study is to select good tools to identify NAFLD from the body composition, anthropometry and related routine clinical parameters. A total of 5076 steelworkers, aged 22–60 years, was included in this study. Body fat mass was measured via bioelectrical impedance analysis (BIA) and fat mass index (FMI) was derived. Ultrasonography method was used to detect hepatic steatosis. Random forest classifier and best subset regression were used to select useful parameters or models that can accurately identify NAFLD. Receiver operating characteristic (ROC) curves were used to describe and compare the performance of different diagnostic indicators and algorithms including fatty liver index (FLI) and hepatic steatosis index (HSI) in NAFLD screening. ROC analysis indicated that FMI can be used with high accuracy to identify heavy steatosis as determined by ultrasonography in male workers [area under the curve (AUC) 0.95, 95% CI 0.93–0.98, sensitivity 89.0%, specificity 91.4%]. The ability of single FMI to identify NAFLD is no less than that of combination panels, even better than the combination panel of HSI. The best subset regression model that including FMI, waist circumference, and serum levels of triglyceride and alanine aminotransferase has moderate accuracy in diagnosing overall NAFLD (AUC 0.83). FMI and the NAFLD best subset (BIC) score seem to be good tools to identify NAFLD in Chinese steelworkers.

## Introduction

Non-alcoholic fatty liver disease (NAFLD) is increasingly a cause of chronic liver disease worldwide and affects about 30% of population in Mainland China^1^. It is estimated that the total NAFLD cases in China will reach 314.58 million by 2030^2^. To make matters worse, China has the youngest median age of NAFLD worldwide, which implies that China will have to bear the burden of NAFLD progression and related complications in the coming decades^2^. Since the availability of liver biopsy in routine physical examination is limited by its invasive nature, there is a pressing need to develop noninvasive NAFLD biomarkers for health monitoring in China. At present, there are several noninvasive tests and scores used to evaluate hepatic steatosis, including fatty liver index (FLI)^3^, hepatic steatosis index (HSI)^4^, NAFLD liver fat score^5^, SteatoTest^6^, and NAFLD ridge score^7^. Although all of the above scores have moderate or good accuracy to diagnose fatty liver, there are some limitations. The inclusion of unconventional indicators, the absence of anthropometric measurements, and the reliability in different populations all limit the wider clinical use of these scores^8^.

Although obesity is a known risk factor for NAFLD, only a few studies have evaluated the value of obesity indices in screening for NAFLD^9,10^. It should be noted that body mass index (BMI) and waist circumference (WC) are the most frequently used predictors in the assessment of NAFLD^10^. However, BMI does not have the ability to distinguish between body fat mass and lean mass. Notably, the excess accumulation of body fat was felt to play a key role in most obesity-associated adverse health outcomes^11^. The prevalence of lean NAFLD (BMI < 25 kg/m^2^) in the Chinese population was reported to be 10.8%^12^, which implies that BMI may not be the best predictor to identify NAFLD, at least in China. In addition, emerging evidence has shown that body fat distribution may be a more important risk factor, which is responsible for NAFLD and metabolic syndrome^9,10,13^. Moreover, a finding that lends support to the idea that percentage of body fat (BF%) and fat mass index (FMI, the fat mass in kilograms divided by the square of the height in meters) seem to be good tools to identify metabolic syndrome^14^. After body size normalization, FMI can eliminate the effect of height on BF%, which can then be used to describe the distribution of body fat^15^. However, there have been no reports to evaluate the potential of FMI in screening for NAFLD.

In terms of the body fat, dual-energy X-ray absorptiometry (DXA) or computed tomography (CT) are still the gold standards for measuring such indicators^16^. Nevertheless, the above-mentioned gold standards are difficult to be used in large-scale population studies or health monitoring due to factors such as radiation and high costs. In fact, bioelectrical impedance analysis (BIA) method is widely used in clinical practice and epidemiological studies to assess body composition, given its convenience, fast, low cost and excellent correlation with magnetic resonance imaging (MRI) and DXA^17^. In addition, the high accuracy of a BIA device (TANITA BC-532) for predicting BF% in health Chinese adults has been confirmed and the same brand of the device (TANITA BC-420) has been used in the China National Health Survey (CNHS)^17,18^. Therefore, we examined the application value of FMI via BIA method in screening for NAFLD among Chinese steelworkers in north China.

## Methods

### Study design and population

This cross-sectional study reported results from the baseline survey of a Chinese occupational cohort conducted among steelworkers in Tangshan City, Hebei Province in north China. The study design and population have been described in detail in our previous studies^19,20^. The main exclusion criteria were diagnosed or suspected secondary causes of hepatic fat accumulation such as excess alcohol intake, or serum hepatitis B surface antigen-positivity. Those who did not complete the ultrasound examination and (or) blood biochemical test and (or) body composition measurement were excluded. Eventually, a total of 5076 participants were included in the present study. All participants provided written informed consent before taking part in this study. This research was approved by the Ethics Committee of North China University of Science and Technology (No.16040).

### Assessment of NAFLD

The diagnosis of NAFLD have been described in detail in one of our previous studies^19^. In brief, ultrasonography method was used to detect hepatic steatosis^21^. Subsequently, according to the ultrasound imaging criteria^22^, the fatty liver was divided into three grades: grade 1 (light), grade 2 (moderate), and grade 3 (heavy). The final assessment of NAFLD excluded secondary hepatic steatosis, including excess alcohol intake (over 140 g/week for men and 70 g/week for women), hepatitis B infection, hepatitis C infection, autoimmune, celiac disease, genetic disorders such as Wilson’s disease, alpha-1-antitrypsin deficiency liver diseases, hepatic malignancies, hepatobiliary infections, biliary tract, and related medications (tamoxifen, amiodarone, methotrexate, glucocorticoids) based on the above ultrasonography results^23,24^.

### Anthropometric measurements

The values of fat mass and BF% were measured by the Body Composition Analyzer (TANITA BC-420, Japan). The manufacturer’s protocols^18^ and the intra-rater reliability^20^ of the body composition analyzer of the same brand and model have been described in detail elsewhere. The measurement criteria of height, weight, waist circumference and hip circumference (HC) in this study were shown in our previous study^20^. The detailed definitions of BF%, FMI, BMI, waist-to-hip ratio (WHR) and waist-to-height ratio (WHtR) have been elaborated in our previous study^20^. Blood pressure measurements were performed three times five-minute intervals using an electronic sphygmomanometer (OMRON, HBP-1100, China), and the participants were required to rest for more than ten minutes. Finally, the mean was obtained for analysis.

### Measurement of laboratory parameters

Overnight fasting blood samples were drawn for determination of fasting plasma glucose (FPG), total cholesterol (TC), triglycerides (TG), low-density lipoprotein (LDL-C), high-density lipoprotein (HDL-C), alanine aminotransferase (ALT), aminotransferase (AST), γ-glutamyl transferase (GGT), platelet count (PLT), serum uric acid, and albumin. All blood samples were tested in the central laboratory of Tangshan Hongci Hospital Laboratory using automatic biochemical analysers (mindrary, BS-800, China) within four hours.

### Assessment of metabolic comorbidities and alcohol consumption

Metabolic comorbidities mainly include diabetes, hyperuricemia, dyslipidaemia, and hypertension. Drinking status was evaluated from self-reported information, mainly including the amount and frequency of alcohol consumed per week. Those who usually consumed some alcohol at least once a week over the past 12 months were defined as current drinkers. For current drinkers, the frequency of drinking status (days/week), usually the average amount of alcohol consumed (g), and types of beverages were recorded. The definition of diabetes, hyperuricemia, dyslipidaemia and hypertension, and the amount of pure alcohol (g/week) consumed per week are provided in the supplementary materials.

### Statistical analysis

Continuous variables were presented as means and standard deviation (SD) and between-group comparisons were performed using analysis of variance (ANOVA) or Student’s t-test if the data were normally distributed. Otherwise, the median (upper quartile–lower quartile) and Kruskal–Wallis test (or Wilcoxon rank sum test) were used to describe and compare these continuous variables among the various groups. The classification data were presented as numbers and percentages, and the χ^2^ test was used to compare differences among groups. The receiver operating characteristic (ROC) curve analyses were performed to determine the appropriate cutoff points for FMI, WC, WHtR, WHR, BF% and BMI in identifying NAFLD. The area under the receiver operating characteristic curves (AUCs) were used to describe the diagnostic abilities of the different anthropometric measurements, and a nonparametric approach described by DeLong et al. was performed to compare the differences between areas^25^. The random forest classifier was used to rank the relative importance of anthropometric indicators and related routine clinical parameters in identifying NAFLD. The variance inflation factor (VIF) and the conditional index were used to diagnose the collinearity of the candidate variables. After finding serious multicollinearity among candidate variables, cluster analysis was used to cluster the variables. Finally, variables with high representativeness (according to cluster analysis) and relative importance (according to random forest classifier) were used as candidates for the best subset regression. To obtain a linear logit, the linearity of logits of all continuous candidate predictors was ascertained using the Box–Tidwell procedure^26^. According to the results of the procedure, a natural logarithmic conversion of GGT and TG was recommended. Information criteria including Akaike information criterion (AIC) and Bayesian information criterion (BIC) were used to determine the best fit in the best subset regression. The tenfold cross-validation method was used to evaluate the generalization ability of different models. The machine learning library (Python 3) scikit-learn (sklearn) was used for model fitting and evaluation. In order to preliminarily explore the relationship between FMI and liver fibrosis, the restricted cubic spline (RCS) models were used to examine the association of FMI with Fibrosis-4 (FIB-4), and NAFLD fibrosis score (NFS). *P* < 0.05 was regarded as significant for two-sided tests. *P* value for pairwise comparisons was adjusted by Bonferroni correction.

### Ethics approval and consent to participate

All procedures performed in studies involving human participants were in accordance with the ethical standards of the institutional and/or national research committee and with the 1964 Helsinki declaration and its later amendments or comparable ethical standards. This research was approved by the Ethics Committee of North China University of Science and Technology (No. 16040). All participants gave informed consent before taking part in this study.

## Results

### General characteristics of the participants

**Figure 1:**
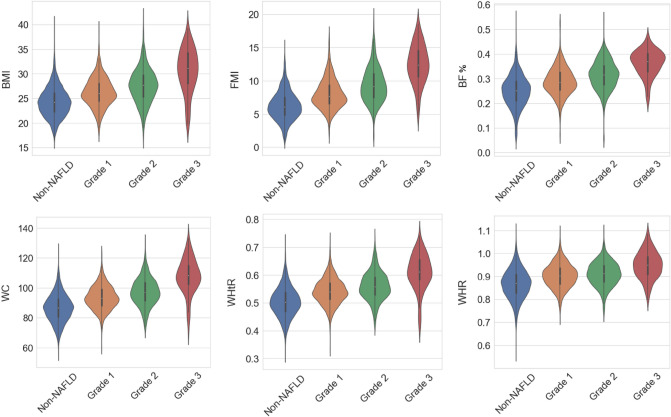
Violin plots of different anthropometric indicators according to grades of steatosis. BMI (kg/m^2^), body mass index; FMI (kg/m^2^), fat mass index; BF%, body fat percentage; WC (cm), waist circumference; WHtR, waist-to-height ratio; WHR, waist-to-height ratio; NAFLD, non-alcoholic fatty liver disease. Grade 1–3 represents the degree of hepatic steatosis.

The general characteristics of the included 5076 participants according to NAFLD status are summarized in Table 1. The general demographic characteristics, including age and sex differed significantly by NAFLD status. No significant differences in ethnicity were observed according to different grades of hepatic steatosis. As for anthropometric indicators, participants with heavy hepatic steatosis (Grade 3) tend to have higher BMI, WC, HC, WHR, WHtR, BF% and FMI. The distribution characteristics of the above indicators in different NAFLD grades are shown in Fig. 1. All of the related routine clinical parameters, including systolic blood pressure (SBP), diastolic blood pressure (DBP), FPG, HDL-C, LDL-C, TG, TC, AST, ALT and GGT differed significantly by grades of hepatic steatosis. With the increase of the degree of hepatic steatosis, the prevalence of diabetes, hypertension, hyperuricemia, and dyslipidaemia also showed an increasing trend. Supplementary Table S1 shows the general characteristics of the study participants according to sex. There were no significant sex discrepancies in age, ethnicity and TC. Compared with female workers, male workers tend to have higher levels of BMI, WC, HC, WHR, WHtR, SBP, DBP, FPG, LDL-C, TG, TC, AST, ALT and GGT, but lower levels of BF%, FMI and HDL-C.

**Table 1:** General characteristics of participants according to grade of hepatic steatosis.

Characteristics	Non-NAFLD	Grade 1	Grade 2	Grade 3	*P* value
	n = 3367	n = 967	n = 658	n = 84	
Age (years), mean ± SD	44.4 ± 7.9	44.2 ± 7.8	43.5 ± 8.0	41.1 ± 8.0	< 0.001
**Sex, n (%)**					< 0.001
Female	354 (10.5)	47 (4.9)	32 (4.9)	2 (2.4)	
Male	3013 (89.5)	920 (95.1)	626 (95.1)	82 (97.6)	
**Ethnicity, n (%)**					0.674
Han	3199 (95.0)	927 (95.9)	625 (95.0)	81 (96.4)	
Others	168 (5.0)	40 (4.1)	33 (5.0)	3 (3.6)	
BMI (kg/m^2^), mean ± SD	24.3 ± 3.0	26.3 ± 3.0	27.6 ± 3.5	30.6 ± 4.5	< 0.001
WC (cm), mean ± SD	86.5 ± 9.3	93.5 ± 8.0	97.3 ± 9.1	107.6 ± 11.5	< 0.001
HC (cm), mean ± SD	99.7 ± 7.0	103.5 ± 6.6	106.9 ± 7.2	113.4 ± 9.4	0.017
WHR, mean ± SD	0.87 ± 0.07	0.90 ± 0.07	0.90 ± 0.05	0.95 ± 0.06	< 0.001
WHtR, mean ± SD	0.50 ± 0.05	0.54 ± 0.05	0.56 ± 0.05	0.61 ± 0.06	< 0.001
BF%, mean ± SD	0.25 ± 0.06	0.29 ± 0.06	0.31 ± 0.06	0.36 ± 0.05	< 0.001
FMI (kg/m^2^), mean ± SD	6.3 ± 2.1	8.0 ± 2.2	9.4 ± 2.6	12.5 ± 2.9	< 0.001
SBP (mmHg), mean ± SD	127.3 ± 15.8	130.4 ± 15.3	132.8 ± 16.2	135.4 ± 15.8	< 0.001
DBP (mmHg), mean ± SD	81.8 ± 10.2	83.5 ± 10.1	83.8 ± 10.3	84.7 ± 11.7	< 0.001
FPG (mmol/L), mean ± SD	5.9 ± 1.1	6.2 ± 1.4	6.3 ± 1.4	6.4 ± 1.3	< 0.001
HDL-C (mmol/L), mean ± SD	1.4 ± 0.3	1.2 ± 0.3	1.2 ± 0.3	1.1 ± 0.3	< 0.001
LDL-C (mmol/L), mean ± SD	3.1 ± 0.8	3.3 ± 0.9	3.3 ± 0.9	3.4 ± 0.9	< 0.001
TG (mmol/L), median (IQR)	1.2 (0.8–1.7)	1.6 (1.1–2.3)	1.8 (1.2–2.7)	1.9 (1.3–2.8)	< 0.001
TC (mmol/L), median (IQR)	5.0 (4.4–5.6)	5.2 (4.6–5.8)	5.3 (4.7–5.9)	5.4 (4.6–6.1)	< 0.001
AST (IU/L), median (IQR)	19.0 (17.0–22.0)	20.0 (18.0–24.0)	21.0 (19.0–26.0)	25.0 (20.0–34.0)	< 0.001
ALT (IU/L), median (IQR)	21.0 (16.0–27.0)	26.0 (20.0–35.0)	31.0 (23.0–44.0)	39.5 (27.0–58.0)	< 0.001
GGT (IU/L), median (IQR)	23.0 (17.0–35.0)	31.0 (22.0–47.0)	36.0 (24.0–51.0)	44.0 (27.5–66.0)	< 0.001
Diabetes, n (%)	208 (6.2)	133 (13.8)	137 (20.8)	19 (22.6)	< 0.001
Hypertension, n (%)	687 (20.4)	284 (29.4)	227 (34.5)	35 (41.7)	< 0.001
Hyperuricemia, n (%)	815 (25.3)	426 (44.1)	350 (53.2)	50 (59.5)	< 0.001
Dyslipidaemia, n (%)	1017 (30.2)	542 (56.1)	438 (66.6)	57 (67.9)	< 0.001

*P-*values are from Pearson’s chi-square test for categorical variables and Kruskal–Wallis test or one-way ANOVA for continuous variables. Grade 1–3 represents the degree of hepatic steatosis. *SD* standard deviation, *IQR* indicates the interquartile range, *BMI* body mass index, *WC* waist circumference, *HC* hip circumference, *WHR* waist-to-height ratio, *WHtR* waist-to-height ratio, *BF%* body fat percentage, *FMI* fat mass index, *SBP* systolic blood pressure, *DBP* diastolic blood pressure, *FGP* fasting plasma glucose, *HDL-C* high-density lipoprotein, *LDL-C* low-density lipoprotein, *TG* triglycerides, *TC* total cholesterol, *AST* aminotransferase, *ALT* alanine aminotransferase, *GGT* γ-glutamyl transferase, *NAFLD* non-alcoholic fatty liver disease.

As shown in Supplementary Table S2, in both genders, all the six anthropometric indicators, including BMI, WC, WHR, WHtR, BF% and FMI were positively correlated with NAFLD. After adjustment for diabetes, dyslipidaemia, hypertension, and hyperuricemia, these estimates were attenuated but remained robust (unadjusted model). Specifically, for per 1 SD increase of FMI, the odds of NAFLD in men and women increased by 2.07-fold (OR 3.07, 95% CI 2.80–3.36) and 1.92-fold (OR 2.92, 95% CI 2.13–4.00), respectively (adjusted model).

### ROC analyses of different anthropometric indicators and combination panels for the screening of NAFLD

Supplementary Figure S1 presents the ROC curves of FMI, WC, WHtR, WHR, BF% and BMI in the total study population, male and female workers respectively, which were used to identify subjects with non-NAFLD from overall NAFLD or different grades of hepatic steatosis. As shown in Supplementary Fig. S1, all the curves were significantly above the diagonal line (all *P* < 0.001). The accuracy of FMI in diagnosing NAFLD was superior to WHtR, WHR, BF%, and BMI [compared with FMI, all *P* values for the difference between AUCs < 0.05/7 (Bonferroni adjusted alpha level)], when the severity of steatosis and sex were not taken into consideration (Table 2). For male workers, FMI remains the optimal anthropometric indicator for the screening of overall NAFLD, with a moderate AUC (95% CI) of 0.78 (0.77–0.80). For female workers, FMI was not superior to abdominal obesity measurements, including WC, WHtR, and WHR, in identifying NAFLD, but it was superior to BMI (*P* = 0.005). When the grades of steatosis was taken into account, the accuracy of FMI in identifying grade 2 or grade 3 steatosis improved in both men and women (women with grade 2 or grade 3 steatosis were combined into one group because of small numbers). In male workers, the optimal cutoff point of FMI in identifying subjects with grade 3 steatosis from non NAFLD was 9.3 kg/m^2^ (according to Youden J-index), with the sensitivity of 89.0%, the specificity of 91.4%, the positive likelihood ratio (+LR) of 10.3, the negative likelihood ratio (−LR) of 0.1, and the AUC (95% CI) of 0.95 (0.93–0.98). The accuracy of FMI in screening for grade 3 steatosis among male workers was superior to WHR (AUC = 0.82, *P* < 0.0001), BF% (AUC = 0.92, *P* < 0.0001) and BMI (AUC = 0.88, *P* = 0.0041), but not superior to WC (AUC = 0.92, *P* = 0.0438, Bonferroni adjusted alpha level was 0.05/7) and WHtR (AUC = 0.90, *P* = 0.0083, Bonferroni adjusted alpha level was 0.05/7). For female workers, the accuracy of FMI in screening for grade 2 or grade 3 steatosis was superior to BF%. The accuracy of FMI in screening for overall NAFLD was superior to the combination panel of HSI (AUC = 0.75, *P* = 0.0026), but not superior to the combination panel of FLI (AUC = 0.79, *P* = 0.3012). For male workers, the accuracy of FMI in identifying overall NAFLD, grade 2 or grade 3 steatosis remained superior to the combination panel of HSI (All *P* for pairwise comparison 0.05/7).

**Table 2:** ROC analyses of different anthropometric indicators and combination panels for the screening of NAFLD.

	Cutoff point	Sensitivity	Specificity	+ LR	− LR	J-value	AUC (95% CI)	*P* value
**All (overall-NAFLD)**								
FMI (kg/m^2^)	7.1	72.0	69.3	2.3	0.4	0.41	0.78 (0.77–0.79)	
WC (cm)	91	71.1	68.3	2.2	0.4	0.39	0.76 (0.75–0.77)	0.0092
WHtR	0.52	72.7	66.1	2.1	0.4	0.39	0.75 (0.74–0.76)	< 0.0001
WHR	0.89	67.4	60.8	1.7	0.5	0.28	0.69 (0.67–0.70)	< 0.0001
BF%	0.27	69.2	64.2	1.9	0.5	0.33	0.73 (0.72–0.74)	< 0.0001
BMI (kg/m^2^)	25.2	70.5	64.2	2.0	0.5	0.35	0.73 (0.72–0.74)	< 0.0001
FLI	48.7	64.5	77.7	2.9	0.5	0.42	0.79 (0.77–0.80)	0.3012
HSI	36.0	67.7	71.8	2.4	0.5	0.39	0.75 (0.74–0.77)	0.0026
**Male (overall-NAFLD)**								
FMI (kg/m^2^)	6.7	78.9	63.2	2.1	0.3	0.42	0.78 (0.77–0.80)	
WC (cm)	91	72.3	66.7	2.2	0.4	0.39	0.76 (0.74–0.77)	< 0.0001
WHtR	0.52	73.0	65.5	2.1	0.4	0.38	0.75 (0.73–0.76)	< 0.0001
WHR	0.89	68.4	58.6	1.7	0.5	0.27	0.68 (0.67–0.69)	< 0.0001
BF%	0.27	68.5	67.0	2.1	0.5	0.35	0.74 (0.73–0.76)	< 0.0001
BMI (kg/m^2^)	25.6	66.8	67.1	2.0	0.5	0.34	0.73 (0.71–0.74)	< 0.0001
FLI	50.2	64.5	77.7	2.9	0.5	0.42	0.78 (0.77–0.79)	0.6300
HSI	36.6	64.4	74.7	2.6	0.5	0.39	0.75 (0.74–0.77)	0.0002
**Female (overall-NAFLD)**								
FMI (kg/m^2^)	8.2	67.9	74.9	2.7	0.4	0.43	0.78 (0.74–0.82)	
WC (cm)	81	91.4	55.4	2.1	0.2	0.47	0.78 (0.74–0.82)	0.9355
WHtR	0.49	93.8	55.1	2.1	0.1	0.49	0.78 (0.74–0.82)	0.9749
WHR	0.81	91.4	44.5	1.6	0.2	0.36	0.73 (0.68–0.77)	0.1710
BF%	0.33	64.2	75.7	2.6	0.5	0.40	0.72 (0.67–0.76)	< 0.0001
BMI (kg/m^2^)	25.8	49.4	82.5	2.8	0.6	0.32	0.70 (0.65–0.74)	0.0050
FLI	22.1	77.8	75.1	3.1	0.3	0.53	0.81 (0.77–0.85)	0.2715
HSI	34.4	70.4	70.6	2.4	0.4	0.41	0.73 (0.68–0.77)	0.0873
**Male (Grade 1)**								
FMI (kg/m^2^)	6.3	81.1	55.2	1.8	0.3	0.36	0.73 (0.72–0.75)	
WC (cm)	88	78.8	53.7	1.7	0.4	0.33	0.71 (0.70–0.73)	0.0187
WHtR	0.52	72.7	60.4	1.8	0.5	0.33	0.71 (0.69–0.72)	0.0028
WHR	0.89	64.8	59.2	1.6	0.6	0.24	0.66 (0.64–0.67)	< 0.0001
BF%	0.25	79.5	50.2	1.6	0.4	0.30	0.70 (0.68–0.71)	< 0.0001
BMI (kg/m^2^)	24.7	73.9	55.6	1.7	0.5	0.30	0.69 (0.67–0.70)	< 0.0001
FLI	47.9	59.5	75.0	2.4	0.5	0.35	0.74 (0.72–0.75)	0.6589
HSI	36.0	61.2	70.6	2.1	0.6	0.32	0.71 (0.69–0.72)	0.0172
**Male (Grade 2)**								
FMI (kg/m^2^)	7.2	80.8	71.5	2.8	0.3	0.52	0.84 (0.82–0.85)	
WC (cm)	92	75.4	71.5	2.7	0.3	0.47	0.80 (0.79–0.81)	0.0001
WHtR	0.52	80.0	65.5	2.3	0.3	0.46	0.79 (0.77–0.80)	< 0.0001
WHR	0.88	71.6	57.7	1.7	0.5	0.29	0.69 (0.68–0.71)	< 0.0001
BF%	0.29	66.5	77.7	3.0	0.4	0.44	0.79 (0.78–0.80)	< 0.0001
BMI (kg/m^2^)	26.7	62.5	79.6	3.1	0.5	0.42	0.77 (0.76–0.78)	< 0.0001
FLI	49.3	74.1	76.8	3.2	0.3	0.51	0.82 (0.81–0.84)	0.2329
HSI	37.8	66.9	81.9	3.7	0.4	0.49	0.80 (0.79–0.82)	0.0046
**Male (Grade 3)**								
FMI (kg/m^2^)	9.3	89.0	91.4	10.3	0.1	0.80	0.95 (0.93–0.98)	
WC (cm)	100	84.2	91.9	10.4	0.2	0.76	0.92 (0.87–0.96)	0.0438
WHtR	0.56	85.4	86.3	6.2	0.2	0.72	0.90 (0.85–0.94)	0.0083
WHR	0.90	81.7	70.2	2.7	0.3	0.52	0.82 (0.77–0.86)	< 0.0001
BF%	0.32	84.2	89.9	8.3	0.2	0.74	0.92 (0.89–0.95)	< 0.0001
BMI (kg/m^2^)	27.8	78.1	88.5	6.8	0.3	0.67	0.88 (0.82–0.93)	0.0041
FLI	64.3	85.4	87.9	7.0	0.2	0.73	0.92 (0.91–0.93)	0.1260
HSI	40.8	72.0	92.8	9.9	0.3	0.65	0.88 (0.86–0.89)	0.0057
**Female (Grade 1)**								
FMI (kg/m^2^)	7.3	76.6	61.3	2.0	0.4	0.40	0.72 (0.65–0.80)	
WC (cm)	83	85.1	61.6	2.2	0.2	0.47	0.76 (0.70–0.81)	0.3794
WHtR	0.49	93.6	53.1	2.0	0.1	0.47	0.76 (0.70–0.82)	0.2729
WHR	0.81	89.4	44.4	1.6	0.2	0.34	0.70 (0.64–0.77)	0.6741
BF%	0.33	51.1	75.7	2.1	0.7	0.27	0.65 (0.57–0.74)	< 0.0001
BMI (kg/m^2^)	23.9	66.0	64.7	1.9	0.5	0.31	0.66 (0.57–0.75)	0.1043
FLI	17.0	78.7	67.2	2.4	0.3	0.46	0.78 (0.74–0.82)	0.1233
HSI	34.4	63.8	70.6	2.2	0.5	0.34	0.68 (0.63–0.73)	0.2925
**Female (Grade 2–3)**								
FMI (kg/m^2^)	9.7	64.7	90.4	6.7	0.4	0.55	0.86 (0.82–0.89)	
WC (cm)	89	70.6	80.5	3.6	0.4	0.51	0.82 (0.75–0.88)	0.2325
WHtR	0.50	94.1	58.8	2.3	0.1	0.53	0.80 (0.74–0.88)	0.0610
WHR	0.87	70.6	72.6	2.6	0.4	0.43	0.76 (0.69–0.84)	0.0391
BF%	0.33	82.4	76.0	3.4	0.2	0.58	0.81 (0.73–0.89)	0.0027
BMI (kg/m^2^)	25.8	58.8	82.5	3.4	0.5	0.41	0.75 (0.65–0.84)	0.0080
FLI	24.1	88.2	77.7	4.0	0.2	0.66	0.85 (0.81–0.89)	0.8390
HSI	36.4	70.6	83.1	4.2	0.4	0.54	0.79 (0.75–0.83)	0.1173

Grade 1–3 represents the degree of hepatic steatosis. *+ LR* positive likelihood ratio, *− LR* negative likelihood ratio, *J-value* Youden J-index (Sensitivity + Specificity − 1), *AUC* area under the receiver operating characteristic curves, *BMI (kg/m*^*2*^*)* body mass index, *FMI (kg/m*^*2*^*)* fat mass index, *BF%* body fat percentage, *WC (cm)* waist circumference, *WHtR* waist-to-height ratio, *WHR* waist-to-height ratio, *NAFLD* non-alcoholic fatty liver disease, *FLI* fatty liver index, *HIS* hepatic steatosis index. *P* values are the significance of the difference in AUC from FMI and WC, WHtR, WHR, BF%, BMI, FLI, HSI by the method described by DeLong et al.

### Anthropometric indicators combined with related routine clinical parameters for NAFLD prediction

**Figure 2:**
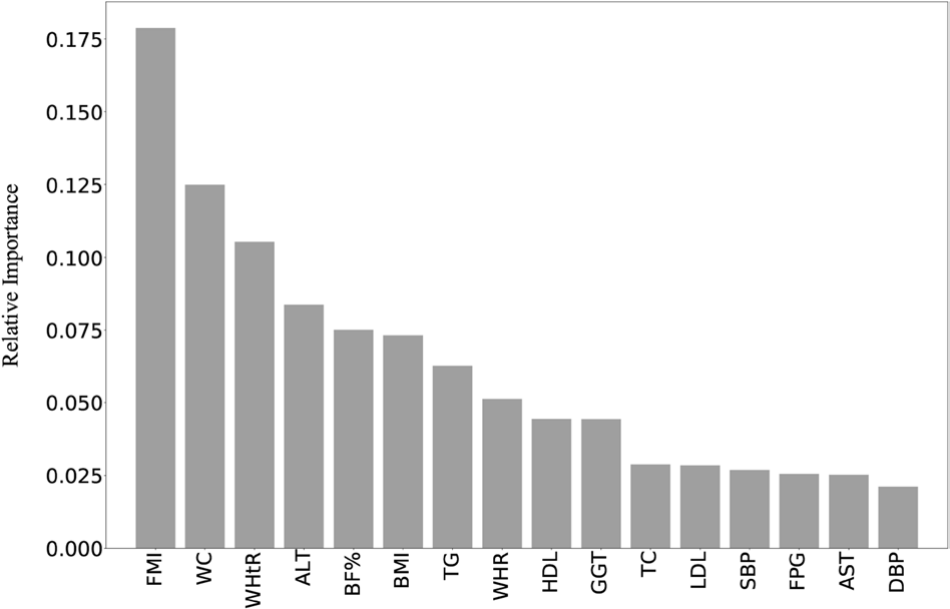
The relative importance of anthropometric and related routine clinical parameters for classification of NAFLD from random forest model. The main parameters determined by grid search: n_estimators = 900, oob_score = True, criterion = 'entropy', max_depth = 9, min_samples_split = 19, min_samples_leaf = 1. *BMI (kg/m*^*2*^*)* body mass index, *FMI (kg/m*^*2*^*)* fat mass index, *BF%* body fat percentage, *WC (cm)* waist circumference, *WHtR* waist-to-height ratio, *WHR* waist-to-height ratio.

The grid search method was used to determine the parameters of the random forest model. Figure 2 shows the relative importance of anthropometric and related routine clinical parameters for classification of NAFLD from random forest model. FMI ranked first in relative importance, followed by WC, WHtR, ALT, BF%, BMI, TG, WHR, HDL, GGT, TC, LDL, SBP, FPG, AST and DBP, respectively. As shown in Supplementary Table S3, collinearity diagnosis results showed severe multicollinearity among the 16 variables in Fig. 2 (conditional index 327.6). Subsequently, cluster analysis was conducted on these 16 variables, and the results were shown in Supplementary Table S4 and Supplementary Fig. S2. Seven variables, including FMI, WC, ALT, TC, SBP, TG, and FPG were selected from each cluster as candidates for the best subset model based on their relative importance to the NAFLD. When age, sex and the above seven variables were included as the candidate variables into the best subset generalized linear model (the information criterion was BIC), the results shown that the model including WC, FMI, ln (TG), and ln (ALT) was the best model (Supplementary Table S5). Supplementary Figure S3 shows the partial nomogram that can be used to manually obtain predicted values of NAFLD from the best subset regression model.

### Model evaluation

We randomly divided 5076 subjects in the present study into training (70%) and validation (30%) groups. Four predicting parameters [WC, FMI, ln (TG), and ln (ALT)] selected from the best subset model were used to fit the model in the training group. The AUCs of the fitted model in training and validation groups to diagnose NAFLD were 0.826 and 0.823, respectively. In the subsequent analysis, we replace the information criterion from BIC to AIC to fit the best subset model. In addition, variables in FLI and HSI were used to fit the logistic regression model (Supplementary Table S5). The model comparison results shown that the accuracy of the best subset model (AUC 0.826, 95% CI 0.815–0.836) (AIC) was superior to FLI model (AUC 0.797, 95% CI 0.786–0.808) and HSI model (AUC 0.750, 95% CI 0.738–0.762), with *P* values for pairwise comparisons < 0.0001 (Supplementary Table S4). No significant difference was observed between the AUCs of the two best subset models (BIC and AIC). In addition, we performed tenfold cross-validation on the above four models. The results shown that the AUCs of the best subset (BIC), best subset (AIC), FLI, and HSI model were 0.823, 0.824, 0.789, and 0.751, respectively, which were comparable with the results in Supplementary Table S5. The best subset (BIC) model was selected as the optimal model in this study, due to its higher accuracy and feasibility in diagnosing NAFLD. According to the regression coefficients summarized in Supplementary Table S5, the equation to calculate NAFLD best subset (BIC) score was the following:

$$\text{NAFLD best subset }\left(\text{BIC}\right)\text{ score}=\frac{1}{1+{e}^{-(-10.95+0.05WC+0.303FMI+0.596\text{ln}\left(TG\right)+1.013\text{ln}(ALT))}}.$$

Supplementary Figure S4 shows the calibration curve of the best subset (BIC) model. No serious deviation from the calibration results was observed, with the Brier score of 0.16. In order to maximize the corresponding specificity and sensitivity respectively, dual cut-offs were selected to achieve 90% sensitivity and 90% specificity to rule out and rule in NAFLD patients. Dual cut-offs of 0.19 and 0.53 were determined for NAFLD best subset (BIC) score to achieve 90% (88–91%) sensitivity and 90% (88–91%) specificity, with meaningful negative predictive value (NPV) of 91% (90–92%) and positive predictive value (PPV) of 72% (69–74%). The dual cut-offs ruled in and ruled out NAFLD in 3211 (63%) subjects. The diagnosis of NAFLD was indeterminate in 1865 (37%) subjects with NAFLD best subset (BIC) scores ranging from 0.19 to 0.53.

### The association of FMI and NAFLD best subset (BIC) score with existing noninvasive biomarkers or panels associated with hepatic steatosis and fibrosis

**Figure 3:**
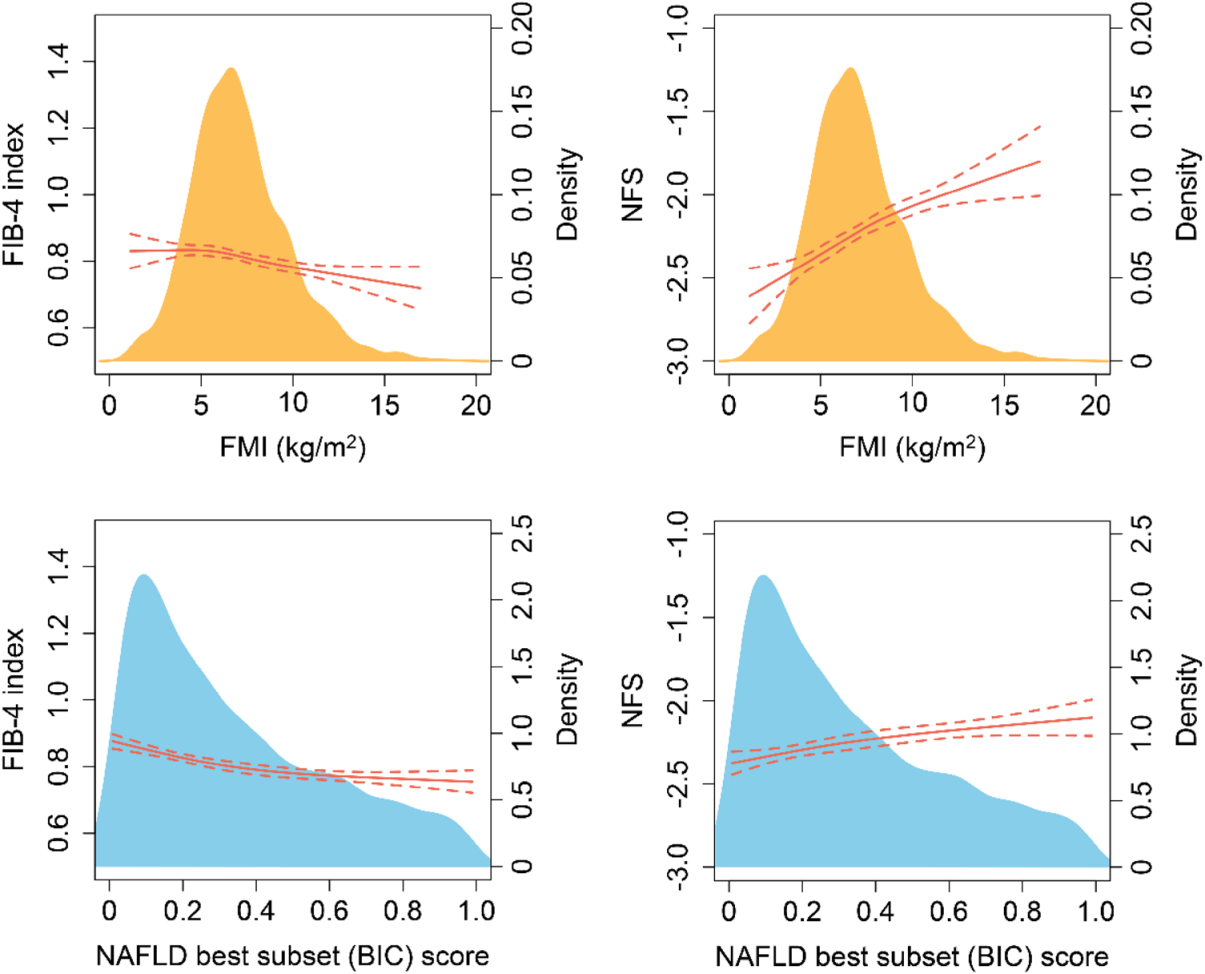
Association of FMI and NAFLD best subset (BIC) score with noninvasive panels associated with liver fibrosis according to restricted cubic spline models. The red dashed line represents the upper and lower bounds of 95% confidence intervals.

The lack of invasive liver biopsy made it difficult to identify nonalcoholic steatohepatitis (NASH) in the present study population, an active histological phenotype of NAFLD with hepatic inflammation and faster fibrosis progression. As for liver fibrosis, FIB-4 index and the NFS are the 2 most popular noninvasive panels for widespread fibrosis screening. In this study, the values of the continuous variables FIB-4 index and NFS were used as proxies to measure the stage of liver fibrosis, and the RCS models showed a significant positive correlation of FMI and NAFLD best subset (BIC) score with NFS (*P* for overall association < 0.05) (Fig. 3). Finally, we calculated the HSI and FLI scores of the present study population separately, following the published algorithms, and compared the screening performance of a single FMI with the combination panels of liver steatosis HSI and FLI. Supplementary Figure S5 presents the ROC curves of FMI, HSI and FLI to identify subjects with non-NAFLD from overall NAFLD or light (grade 1) to heavy (grade 3) degree of hepatic steatosis. As shown in Supplementary Table S6, the performance of FMI and FLI was almost comparable in identifying hepatic steatosis (*P* for pairwise comparison > 0.05/3). While, the single FMI performs better than the combination panel of HSI in identifying hepatic steatosis (*P* for pairwise comparison < 0.05/3).

In subsequent analyses, we separately analyzed the performance of FMI in identifying hepatic steatosis in different BMI subgroups. The results showed that FMI was better at identifying hepatic steatosis in subjects with a BMI of less than 25 kg/m^2^ than in those with a BMI of 25 to 30 kg/m^2^ (Supplementary Table S7).

## Discussion

In the present study, we showed that FMI and NAFLD best subset (BIC) score were useful tools for the assessment of NAFLD. For male workers, FMI had very good accuracy to distinguish subjects with heavy hepatic steatosis from non-NAFLD subjects. The NAFLD best subset (BIC) score that combines anthropometric indicators and common clinical parameters which can be easily available in the regular health check-up, showed a moderate performance in screening for overall NAFLD. At the dual cut-offs of 0.19 and 0.53, NAFLD best subset (BIC) score achieved 90% sensitivity and 90% specificity in the study population with 91% NPV and 72% PPV, respectively.

Several studies have evaluated the role of body composition and abdominal obesity indicators in screening for NAFLD^9,10^. In line with previous studies, we showed that the simple anthropometric parameter WC is a useful tool for identifying NAFLD^9,10,27^. In addition, a previous study on the relationship between body composition variables and NAFLD indicated that intra-abdominal adipose tissue (diagnosed by ultrasound) was also a potential predictor of NAFLD^10^. The BF% and FMI (measured by bioelectrical impedance analysis) have been reported as good tools to identify metabolic syndrome^14,28^, which is closely associated with NAFLD^29^. However, evidence regarding the performance of BF% and FMI in identifying NAFLD remains sparse. Several prediction index and scores have been developed to identify NAFLD^8^. Given the accessibility of electronic health records, many existing NAFLD prediction models make full use of laboratory parameters as predictors, while anthropometric measurements are often missing. For example, NAFLD liver fat score^5^ and NAFLD ridge score^7^ were only dependent on laboratory biomarkers, which may limit their ability to identify NAFLD. Aside from that, some parameters, such as insulin and glycosylated haemoglobin (HbA_1c_) are not always routinely measured, which will lead to their limited availability. Notably, among those models that involved both laboratory and anthropometric parameters, BMI was the most commonly used anthropometric index^3,4,6^. Although about 80% of patients with NAFLD are obese (BMI > 30 kg/m^2^), BMI does not differentiate between body lean mass and body fat mass^30^. Moreover, the prevalence of lean NAFLD (BMI < 25 kg/m^2^) in the Chinese population was reported to be 10.8%^12^, which implies that the accuracy of BMI in predicting NAFLD may be limited. Stranges et al.^13^ concluded that BMI was not a reliable marker of fatty liver. While, FMI, an alternative simple and inexpensive approach for assessing body fat distribution, has been reported as a surrogate marker of cardiovascular risk and metabolic syndrome^14,15^. Our present study aligns with the idea that the distribution of fat tissue (assessed by FMI) plays a greater role in NAFLD than the BMI^30^. Furthermore, this study adds to the evidence that FMI is not only strongly associated with overall NAFLD, but also with the degree of steatosis. The results of model comparison shown that the accuracy of the best subset model in the diagnosis of NAFLD was better than that of FLI and HSI models in our study population. The main difference between the current model and the previous models is the inclusion of FMI as a predictive indicator, which indirectly supports the important role of FMI in identifying NAFLD, at least among Chinese steelworkers in north China.

In our NAFLD best subset (BIC) score, anthropometric indicators include WC in addition to FMI. This is consistent with previous studies showing that abdominal fat accumulation can be an independent predictor of hepatic steatosis^13^. In particular, a study in Korean reported that WC was as useful as DXA and CT in screening for NAFLD^9^. However, WC suffered from a key limitation in that it cannot differentiate between visceral and subcutaneous fat. While, what we already known is that visceral adipose tissue is directly associated with the development and progression of hepatic steatosis^30^. Therefore, the role of FMI cannot be completely replaced. Interestingly, pairwise comparison of ROC show that the performance of single FMI to identify hepatic steatosis is no less than that of combination panels and even better than the combination panel of HSI. Overall, the role of FMI in identifying liver steatosis may be more practical in terms of practical availability and feasibility. As for the laboratory indicators, the logarithmic conversion values of TG and ALT are included in our best subset model. In theory, excess free fatty acids are considered to be one of the most important factors contributing to the development and progression of NAFLD. NAFLD arises when the uptake of fatty acids and TG from circulation and de novo lipogenesis saturate the rate of fatty acids β-oxidation and very-low density lipoprotein (VLDL)-TG export^31^. Therefore, it seems logical to use serum levels of TG to screen for NAFLD. Evidence is particularly extensive with regard to the link between liver enzymes and NAFLD. Elevated liver enzymes were reported in about 20% of patients with NAFLD^32^. The liver enzymes included in previous models for predicting NAFLD were mainly ALT, AST, GGT and AST/ALT ratio. Among these liver enzymes, ALT serves as a specific marker of liver inflammation and hepatocellular injury^33^. Differences in composition of NAFLD subtypes between different studies were thus more likely to contribute to the discrepancy in selection of liver enzymes in different NAFLD screening models.

On the basis of disease severity, NAFLD is divided into nonalcoholic fatty liver (NAFL) and NASH. NASH is the active form of NAFLD characterized by histological lobular inflammation and hepatocyte ballooning and is associated with cirrhosis, hepatocellular carcinoma, liver transplantation, and death. Although FMI performs better in identifying liver steatosis, the significance of detecting liver inflammation and fibrosis in clinical practice may be of greater concern^34^. Unfortunately, the lack of liver biopsy made it difficult to diagnose NASH directly. However, to achieve better diagnostic of liver fibrosis through noninvasive methods, many biomarkers and panels have been developed, among which FIB-4 index and the NFS are the 2 most popular noninvasive panels for fibrosis screening^35^. We observed that FMI was positively associated with NFS, but not FIB4-4. For FIB-4, dual cutoffs of < 1.45 and of > 3.25 were used to rule-out and rule-in elevated liver stiffness^36^. In other words, individuals with FIB-4 values inside 1.45–3.25 would not be correctly classified. Therefore, the relationship between FMI and FIB-4 may be affected by individuals with FIB-4 values inside 1.45–3.25. Given that indirect biomarkers are in general less accurate than biomarkers directly measuring fibrogenesis or fibrinolysis, the reproducibility of FIB-4 in different populations needs to be further verified. It is noteworthy, however, that the NFS was specifically derived and validated in patients with biopsy-proven NAFLD, and thus may have a higher accuracy. In addition, NFS has been well validated in Chinese populations^37^. Although this study failed to directly define the role of FMI in the detection of liver fibrosis, the relationship between FMI and NFS, a proxy of fibrosis, supports the potential research value of FMI in this area.

The major strengths of our study include the large sample size, good availability and inexpensive screening indicators. To our knowledge, this is the first study to explore the usefulness of FMI in screening for NAFLD. However, our study also has certain limitations. First, although FMI and NAFLD best subset (BIC) score can be good for detecting NAFLD in cross-sectional study, their accuracy in assessing changes in liver fat over time is still unknown. Second, BIA is a predictive method that requires assumptions based on population mean values. An improved standardization of protocols for measurement is essential^38^. Third, the sample size of females in this study was small, so it was difficult for us to identify sex differences. Although our understanding of sex differences in NAFLD remains insufficient, adequate consideration of sex differences are needed to implement precision medicine for patients with NAFLD. Previous evidence has shown that the prevalence and severity of NAFLD are higher in men than in women during the reproductive age, whereas NAFLD occurs at a higher rate in postmenopausal women, suggesting a protective effect of estrogen^39^. The sex difference may be due to differences in hormone levels. In addition, according to the results in Supplementary Table S1, sex differences may also be related to the discrepancy distribution of general characteristics of male and female workers, despite the limited sample size. Future studies should include sufficient and comparable female subjects to identify sex differences. Fourth, although the dual cut-offs can maximize both sensitivity and specificity in ruling out and ruling in NAFLD patients, the NAFLD best subset (BIC) score has a somewhat low PPV (72%). Fifth, our survey population consisted of steelworkers in north China, which limits our ability to generalize these results to the general population. It seems that FMI and the NAFLD best subset (BIC) score need external validation to widely use. Sixth, the assessment of NAFLD by ultrasound may be subjective and inconsistent. At present, tissue biopsy is still the gold standard for diagnosing NAFLD. However, this method is not feasible in large-scale epidemiological investigations, since most people affected by NAFLD are likely to be asymptomatic, so other noninvasive methods like ultrasonography, is advised and might be preferred^40,41^. Seventh, due to the lack of liver biopsy, it is difficult to directly determine the performance of FMI in screening for liver inflammation and fibrosis. However, the relationship between FMI and proxy metrics of liver inflammation and fibrosis also indirectly supports the potential research value of FMI.

## Conclusion

FMI and NAFLD best subset (BIC) score seem to be good tools for screening of liver steatosis. The NAFLD best subset (BIC) score that combines anthropometric indicators and common clinical parameters which can be easily available in the regular health check-up, is a simple and robust reference to identify overall NAFLD patients among steelworkers in north China.

## Supplementary Information

Supplementary Information.

## Data Availability

The data that support the findings of this study are available from [Institute of basic medicine, Chinese academy of medical sciences] but restrictions apply to the availability of these data, which were used under license for the current study, and so are not publicly available. Data are however available from the authors upon reasonable request. For data request, please contact professor Yuan Juxiang (email address: yuanjx@ncst.edu.cn).

## Abbreviations

NAFLD: Non-alcoholic fatty liver disease
BIA: Bioelectrical impedance analysis
FMI: Fat mass index
ROC: Receiver operating characteristic
OR: Odds ratio
CI: Confidence interval
AUC: Area under the curve
FLI: Fatty liver index
HSI: Hepatic steatosis index
BMI: Body mass index
WC: Waist circumference
BF%: Percentage of body fat
DXA: Dual-energy X-ray absorptiometry
CT: Computed tomography
HC: Hip circumference
WHR: Waist-to-hip ratio
WHtR: Waist-to-height ratio
FPG: Fasting plasma glucose
TC: Total cholesterol
TG: Triglycerides
LDL-C: Low-density lipoprotein
HDL-C: High-density lipoprotein
ALT: Alanine aminotransferase
AST: Aminotransferase
GGT: γ-Glutamyl transferase
SD: Standard deviation
ANOVA: Analysis of variance
AIC: Akaike information criterion
BIC: Bayesian information criterion
SBP: Systolic blood pressure
DBP: Diastolic blood pressure
+LR: Positive likelihood ratio
−LR: Negative likelihood ratio
NASH: Nonalcoholic steatohepatitis
FIB-4: Fibrosis-4 index
NFS: Nonalcoholic fatty liver disease fibrosis score
