# Supplementary material for: Fat mass index as a screening tool for the assessment of non-alcoholic fatty liver disease
Source: Sci Rep. 2022 Nov 23;12:20219. doi: 10.1038/s41598-022-23729-1 (PMC9684573; doi:10.1038/s41598-022-23729-1)
Supplement: Supplementary file 1 — Supplementary Information. [file 41598_2022_23729_MOESM1_ESM.docx]

***Supplementary material：***

**Fat mass index as a screening** **tool** **for the assessment of** **non-alcoholic fatty liver disease**

***Assessment of Covariates***

Elevated blood pressure was defined as current systolic blood pressure ≥140 mmHg, diastolic blood pressure ≥90 mmHg, or if the patient was receiving antihypertensive therapy. Participants were required to fast overnight before the abdominal ultrasound examination and blood collection. Participants’ anterior elbow vein blood was collected and centrifuged at room temperature (3000 r/min, 15 minutes) immediately. All blood samples were tested in the central laboratory of Tangshan Hongci Hospital Laboratory using automatic biochemical analysers (mindrary, BS-800, China) within four hours. Total cholesterol (TC) ≥6.22 mmol/L or low-density lipoprotein (LDL-C) ≥4.11 mmol/L or high-density lipoprotein (HDL-C) ≤1.04 mmol/L or triglycerides (TG) ≥2.32 mmol/L, or patients undergoing lipid-lowering therapy were considered to demonstrate ^1^. Diabetes was defined as fasting blood glucose ≥7.0 mmol/L or if the patient was receiving hypoglycemic therapy. Impaired fasting glucose (IFG) was defined as a fasting glucose between 5.6 and 7.0 mmol/L ^2^. Serum uric acid concentrations greater than 6 mg/dL (357 μmol/L) for females, 7 mg/dL (416 μmol/L) for males, are defined as hyperuricemia ^3^. Participants' drug use history was collected through face-to-face questionnaires. Alcohol content across different types of beverages in China was assessed as follows: strong spirits 53%, weak spirits 38%, rice wine 15%, grape wine 12%, and beer 4%. Based on the beverage type, amount consumed, and frequency, we were able to derive the amount of pure alcohol (g/week) consumed per week.^4^ Intake levels of two standard drinks (20 g ethanol daily, 140 g weekly) daily for males and one standard drink daily (70 g weekly) for females were considered acceptable thresholds for defining non-alcoholics.^5^

***Table of contents***

**Table S1** General characteristics of the study participants according to sex.

**Table S2**. Odds ratio of NAFLD according to 1 SD increase of different anthropometric indicators.

**Table S3** Collinearity diagnosis of candidate variables.

**Table S4** Cluster analysis of candidate variables.

**Table S5** Parameters of different models and comparison between models.

**Table S6** Pairwise comparison of ROC curves.

**Table S7** ROC analyses of FMI for the screening of NAFLD according to different BMI subgroups.

***Figure of contents***

**Fig. S1** ROC curves of different anthropometric indicators for the screening of NAFLD.

**Fig. S2** Horizontal dendrogram of cluster analysis results.

**Fig. S3** The nomogram for NAFLD according to the best subset regression.

**Fig. S4** The calibration curve for the best subset (BIC) model.

**Fig. S5** ROC curves of different combination panels and FMI for the screening of NAFLD.

**Table S1** General characteristics of the study participants according to sex.

| Variables | Total | Female | Male |  |
| --- | --- | --- | --- | --- |
|  | N=5076 | n=435 | n=4641 | *P* value |
| Age (years), mean ± SD | 44.2 ± 8.0 | 44.1 ± 5.0 | 44.2 ± 8.2 | 0.634 |
| Ethnicity, n (%) |  |  |  | 0.798 |
| Han | 4832 (95.2) | 413 (94.9) | 4419 (95.2) |  |
| Others | 244 (4.8) | 22 (5.1) | 222 (4.8) |  |
| BMI (kg/m^2^), mean ± SD | 25.2 ± 3.4 | 23.8 ± 3.2 | 25.4 ± 3.4 | <0.001 |
| WC (cm), mean ± SD | 89.6 ± 10.2 | 83.2 ± 11.0 | 90.2 ± 9.9 | <0.001 |
| HC (cm), mean ± SD | 101.6 ± 7.6 | 98.9 ± 7.5 | 101.8 ± 7.6 | <0.001 |
| WHR, mean ± SD | 0.88 ± 0.07 | 0.84 ± 0.07 | 0.89 ± 0.07 | <0.001 |
| WHtR, mean ± SD | 0.52 ± 0.06 | 0.50 ± 0.06 | 0.52 ± 0.06 | <0.001 |
| BF%, mean ± SD | 26.8 ± 6.8 | 29.1 ± 7.8 | 26.6 ± 6.6 | <0.001 |
| FMI ((kg/m^2^), mean ± SD | 7.1 ± 2.6 | 7.3 ± 2.7 | 7.1 ± 2.6 | 0.058 |
| SBP (mmHg), mean ± SD | 128.7 ± 15.9 | 122.5 ± 14.8 | 129.3 ± 15.9 | <0.001 |
| DBP (mmHg), mean ± SD | 82.4 ± 10.3 | 77.6 ± 9.8 | 82.9 ± 10.2 | <0.001 |
| FPG (mmol/L), mean ± SD | 6.0 ± 1.2 | 5.7 ± 1.0 | 6.1 ± 1.3 | <0.001 |
| HDL-C cholesterol (mmol/L), mean ± SD | 1.3 ± 0.3 | 1.5 ± 0.4 | 1.3 ± 0.3 | <0.001 |
| LDL-C cholesterol (mmol/L), mean ± SD | 3.2 ± 0.9 | 3.1 ± 0.9 | 3.2 ± 0.9 | 0.029 |
| TG (mmol/L), median (IQR) | 1.30 (0.90–1.95) | 0.95 (0.73–1.40) | 1.34 (0.93–1.99) | <0.001 |
| TC (mmol/L), mean ± SD | 5.14 ± 0.97 | 5.09 ± 0.98 | 5.15 ± 0.96 | 0.230 |
| AST (U/L), median (IQR) | 20.0 (17.0–23.0) | 18.0 (16.0–21.0) | 20.0 (17.0–23.0) | <0.001 |
| ALT (U/L), median (IQR) | 23.0 (17.0–31.0) | 17.0 (13.0–22.0) | 23.0 (18.0–32.0) | <0.001 |
| GGT (U/L), median (IQR) | 26.0 (18.0–41.0) | 15.0 (12.0–21.0) | 27.0 (19.0–42.0) | <0.001 |

*P-*values are from Pearson’s chi-square test for categorical variables and Student’s t-test or Wilcoxon rank sum test for continuous variables. SD, standard deviation; IQR, indicates the interquartile range; BMI, body mass index; WC, waist circumference; HC, hip circumference; WHR, waist-to-height ratio; WHtR, waist-to-height ratio; BF%, body fat percentage; FMI, fat mass index; SBP, systolic blood pressure; DBP, diastolic blood pressure; FGP, fasting plasma glucose; HDL-C, high-density lipoprotein; LDL-C, low-density lipoprotein; TG, triglycerides; TC, total cholesterol; AST, aminotransferase; ALT, alanine aminotransferase; GGT, γ-glutamyl transferase.

**Table S2**. Odds ratio of NAFLD according to 1 SD increase of different anthropometric indicators

| Variable | Unadjusted model | | |  | Adjusted model | | |
| --- | --- | --- | --- | --- | --- | --- | --- |
|  | *β* | OR (95% CI) | *P*-value |  | *β* | OR (95% CI) | *P*-value |
| Male |  |  |  |  |  |  |  |
| FMI (kg/m^2^) | 1.24 | 3.44 (3.16–3.76) | <0.001 |  | 1.12 | 3.07 (2.80–3.36) | <0.001 |
| WC (cm) | 1.10 | 3.00 (2.76–3.26) | <0.001 |  | 0.98 | 2.66 (2.44–2.90) | <0.001 |
| WHtR | 1.02 | 2.76 (2.55–2.99) | <0.001 |  | 0.91 | 2.48 (2.28–2.69) | <0.001 |
| WHR | 0.70 | 2.02 (1.87–2.19) | <0.001 |  | 0.57 | 1.76 (1.62–1.92) | <0.001 |
| BF% | 1.02 | 2.79 (2.57–3.02) | <0.001 |  | 0.91 | 2.48 (2.28–2.71) | <0.001 |
| BMI (kg/m^2^) | 0.90 | 2.45 (2.27–2.64) | <0.001 |  | 0.80 | 2.23 (2.06–2.41) | <0.001 |
| Female |  |  |  |  |  |  |  |
| FMI (kg/m^2^) | 1.11 | 3.02 (2.26–4.04) | <0.001 |  | 1.07 | 2.92 (2.13–4.00) | <0.001 |
| WC (cm) | 0.91 | 2.50 (1.94–3.21) | <0.001 |  | 0.83 | 2.28 (1.74–3.00) | <0.001 |
| WHtR | 0.87 | 2.38 (1.86–3.04) | <0.001 |  | 0.84 | 2.32 (1.77–3.05) | <0.001 |
| WHR | 0.82 | 2.28 (1.74–2.99) | <0.001 |  | 0.71 | 2.04 (1.53–2.73) | <0.001 |
| BF% | 0.78 | 2.18 (1.66–2.86) | <0.001 |  | 0.81 | 2.24 (1.67–3.01) | <0.001 |
| BMI (kg/m^2^) | 0.77 | 2.15 (1.65–2.80) | <0.001 |  | 0.65 | 1.91 (1.44–2.53) | <0.001 |

*β*, regression coefficient; SE, standard error; OR, odds ratio; CI, confidence interval; BMI (kg/m2), body mass index; FMI (kg/m2), fat mass index; BF%, body fat percentage; WC (cm), waist circumference; WHtR, waist-to-height ratio; WHR, waist-to-height ratio. Adjusted for diabetes, dyslipidaemia, hypertension, and hyperuricemia.

**Table S3** Collinearity diagnosis of candidate variables.

| Variable | VIF | |
| --- | --- | --- |
|  | Before cluster ^a^ | After cluster ^b^ |
| FMI | 16.3 | 1.8 |
| WC | 10.7 | 1.8 |
| ALT | 3.0 | 1.1 |
| TC | 32.0 | 1.1 |
| TG | 12.1 | 1.2 |
| SBP | 2.0 | 1.1 |
| FPG | 1.1 | 1.1 |
| BMI | 2.2 |  |
| WHR | 2.1 |  |
| WHtR | 9.6 |  |
| BF% | 11.3 |  |
| AST | 3.1 |  |
| GGT | 1.5 |  |
| DBP | 1.9 |  |
| HDL-C | 4.5 |  |
| LDL-C | 26.1 |  |

^a^ Conditional index is 327.6; ^b^ Conditional index is 6.3; VIF, variance inflation factor ;BMI, body mass index; WC, waist circumference; HC, hip circumference; WHR, waist-to-height ratio; WHtR, waist-to-height ratio; BF%, body fat percentage; FMI, fat mass index; SBP, systolic blood pressure; DBP, diastolic blood pressure; FGP, fasting plasma glucose; HDL-C, high-density lipoprotein; LDL-C, low-density lipoprotein; TG, triglycerides; TC, total cholesterol; AST, aminotransferase; ALT, alanine aminotransferase; GGT, γ-glutamyl transferase.

**Table S4** Cluster analysis of candidate variables.

| Clusters | Variable | R^2^ with | | 1-R^2^ Ratio |
| --- | --- | --- | --- | --- |
|  |  | Own Cluster | Next Closest |  |
| Cluster 1 | BMI | 0.583 | 0.248 | 0.555 |
|  | BF | 0.865 | 0.239 | 0.178 |
|  | FMI | 0.941 | 0.364 | 0.093 |
| Cluster 2 | AST | 0.851 | 0.013 | 0.151 |
|  | GGT | 0.511 | 0.038 | 0.508 |
|  | ALT | 0.767 | 0.065 | 0.249 |
| Cluster 3 | TC | 0.908 | 0.024 | 0.094 |
|  | LDL-C | 0.908 | 0.019 | 0.094 |
| Cluster 4 | SBP | 0.842 | 0.047 | 0.166 |
|  | DBP | 0.842 | 0.017 | 0.161 |
| Cluster 5 | WC | 0.918 | 0.421 | 0.141 |
|  | WHR | 0.715 | 0.106 | 0.319 |
|  | WHtR | 0.914 | 0.416 | 0.148 |
| Cluster 6 | TG | 0.673 | 0.056 | 0.346 |
|  | HDL-C | 0.673 | 0.076 | 0.354 |
| Cluster 7 | FPG | 1.000 | 0.030 | 0.000 |

Total variation explained = 12.909, proportion = 0.807. BMI, body mass index; WC, waist circumference; HC, hip circumference; WHR, waist-to-height ratio; WHtR, waist-to-height ratio; BF%, body fat percentage; FMI, fat mass index; SBP, systolic blood pressure; DBP, diastolic blood pressure; FGP, fasting plasma glucose; HDL-C, high-density lipoprotein; LDL-C, low-density lipoprotein; TG, triglycerides; TC, total cholesterol; AST, aminotransferase; ALT, alanine aminotransferase; GGT, γ-glutamyl transferase.

**Table S5** Parameters of different models and comparison between models.

| Parameter | *β* | SE (*β*) | *P*-value | AUC (95% CI) |
| --- | --- | --- | --- | --- |
| **Best subset (BIC)-model** |  |  |  | 0.826 (0.815 to 0.836) |
| Intercept | -10.950 | 0.470 | <0.001 |  |
| WC (cm) | 0.050 | 0.005 | <0.001 |  |
| FMI (kg/m^2^) | 0.303 | 0.020 | <0.001 |  |
| ln (TG, mmol/L) | 0.596 | 0.062 | <0.001 |  |
| ln (ALT, U/L) | 1.013 | 0.085 | <0.001 |  |
| **Best subset (AIC)-model** |  |  |  | 0.827 (0.817 to 0.838) ^a^ |
| Intercept | -11.260 | 0.570 | <0.001 |  |
| Age | -0.010 | 0.005 | 0.033 |  |
| Sex | 0.409 | 0.156 | 0.009 |  |
| WC (cm) | 0.047 | 0.005 | <0.001 |  |
| FMI (kg/m^2^) | 0.315 | 0.020 | <0.001 |  |
| FPG (mmol/L) | 0.080 | 0.028 | 0.005 |  |
| TC (mmol/L) | 0.065 | 0.040 | 0.102 |  |
| ln TG | 0.535 | 0.066 | <0.001 |  |
| ln ALT | 0.945 | 0.087 | <0.001 |  |
| **FLI-model** |  |  |  | 0.797 (0.786 to 0.808) ^b^ |
| Intercept | -12.680 | 0.452 | <0.001 |  |
| WC (cm) | 0.082 | 0.004 | <0.001 |  |
| BMI (kg/m^2^) | 0.123 | 0.012 | <0.001 |  |
| ln (TG, mmol/L) | 0.547 | 0.064 | <0.001 |  |
| ln (GGT, U/L) | 0.347 | 0.062 | <0.001 |  |
| **HSI-model** |  |  |  | 0.750 (0.738 to 0.762) ^c^ |
| Intercept | -6.241 | 0.401 | <0.001 |  |
| Sex | 0.330 | 0.139 | 0.018 |  |
| BMI (kg/m^2^) | 0.222 | 0.011 | <0.001 |  |
| FPG (mmol/L) | 0.116 | 0.253 | <0.001 |  |
| AST/ALT ratio | -1.310 | 0.138 | <0.001 |  |

BIC, Bayesian information criterion; AIC, Akaike information criterion; FLI, fatty liver index; HIS, hepatic steatosis index; WC (cm), waist circumference; FMI (kg/m^2^), fat mass index; BMI, body mass index; ln (TG, mmol/L), natural logarithm of serum triglyceride levels; ln (ALT, U/L), natural logarithm of serum alanine aminotransferase levels; FPG, fasting plasma glucose; ln (GGT, U/L), natural logarithm of serum γ-glutamyl transferase levels.

^a^ *P* value of difference between AUCs (Best subset (AIC)-model vs Best subset (BIC)-model) = 0.0632.

^b^ *P* value of difference between AUCs (FLI-model vs Best subset (BIC)-model) < 0.0001.

^c^ *P* value of difference between AUCs (HSI-model vs Best subset (BIC)-model) < 0.0001.

**Table S6** Pairwise comparison of ROC curves.

| Biomarker | Overall | |  | Moderate to heavy hepatic steatosis | |
| --- | --- | --- | --- | --- | --- |
|  | AUC (95% CI) | *P* for pairwise comparison |  | AUC (95% CI) | *P* for pairwise comparison |
| FMI | 0.777 (0.766 to 0.789) | FMI vs FLI: *P*=0.301 |  | 0.845 (0.833 to 0.855) | FMI vs FLI: *P*=0.449 |
| FLI | 0.785 (0.773 to 0.796) | FLI vs HSI: *P*<0.001 |  | 0.838 (0.826 to 0.849) | FLI vs HSI: *P*=0.001 |
| HSI | 0.754 (0.741 to 0.765) | HSI vs FMI: *P*=0.003 |  | 0.813 (0.800 to 0.825) | HSI vs FMI: *P*=0.002 |

FMI (kg/m^2^), fat mass index; FLI, fatty liver index; HSI, hepatic steatosis index. *P* values are the significance test of difference between AUCs.

**Table S7** ROC analyses of FMI for the screening of NAFLD according to different BMI subgroups.

| Subgroup | Cutoff point | Sensitivity | Specificity | +LR | −LR | J-value | AUC (95% CI) |
| --- | --- | --- | --- | --- | --- | --- | --- |
| BMI<25 (kg/m^2^) |  |  |  |  |  |  |  |
| Overall-NAFLD | 6.3 | 66.3 | 71.0 | 2.3 | 0.5 | 0.37 | 0.75 (0.73 to 0.77) |
| Grade 1 | 6.3 | 63.6 | 71.0 | 2.2 | 0.5 | 0.35 | 0.73 (0.71 to 0.74) |
| Grade 2–3 | 6.1 | 79.7 | 65.1 | 2.3 | 0.3 | 0.45 | 0.80 (0.78 to 0.82) |
| 25≤BMI<30 (kg/m^2^) |  |  |  |  |  |  |  |
| Overall-NAFLD | 7.9 | 60.0 | 62.9 | 1.6 | 0.6 | 0.23 | 0.66 (0.64 to 0.68) |
| Grade 1 | 6.7 | 82.2 | 35.4 | 1.3 | 0.5 | 0.18 | 0.61 (0.59 to 0.63) |
| Grade 2–3 | 7.9 | 71.2 | 63.6 | 2.0 | 0.5 | 0.35 | 0.73 (0.70 to 0.75) |
| BMI>30 (kg/m^2^) |  |  |  |  |  |  |  |
| Overall-NAFLD | 9.7 | 82.9 | 64.4 | 2.3 | 0.3 | 0.47 | 0.79 (0.74 to 0.82) |
| Grade 1 | 9.7 | 71.3 | 64.4 | 2.0 | 0.5 | 0.36 | 0.70 (0.63 to 0.76) |
| Grade 2–3 | 9.7 | 88.7 | 64.4 | 2.5 | 0.2 | 0.53 | 0.83 (0.78 to 0.87) |

FMI (kg/m^2^), fat mass index; BMI, body mass index; Grade 1 to Grade 3 represents the degree of hepatic steatosis. +LR, positive likelihood ratio; −LR, negative likelihood ratio; J-value, Youden J-index (Sensitivity + Specificity - 1); AUC, area under the receiver operating characteristic curves.


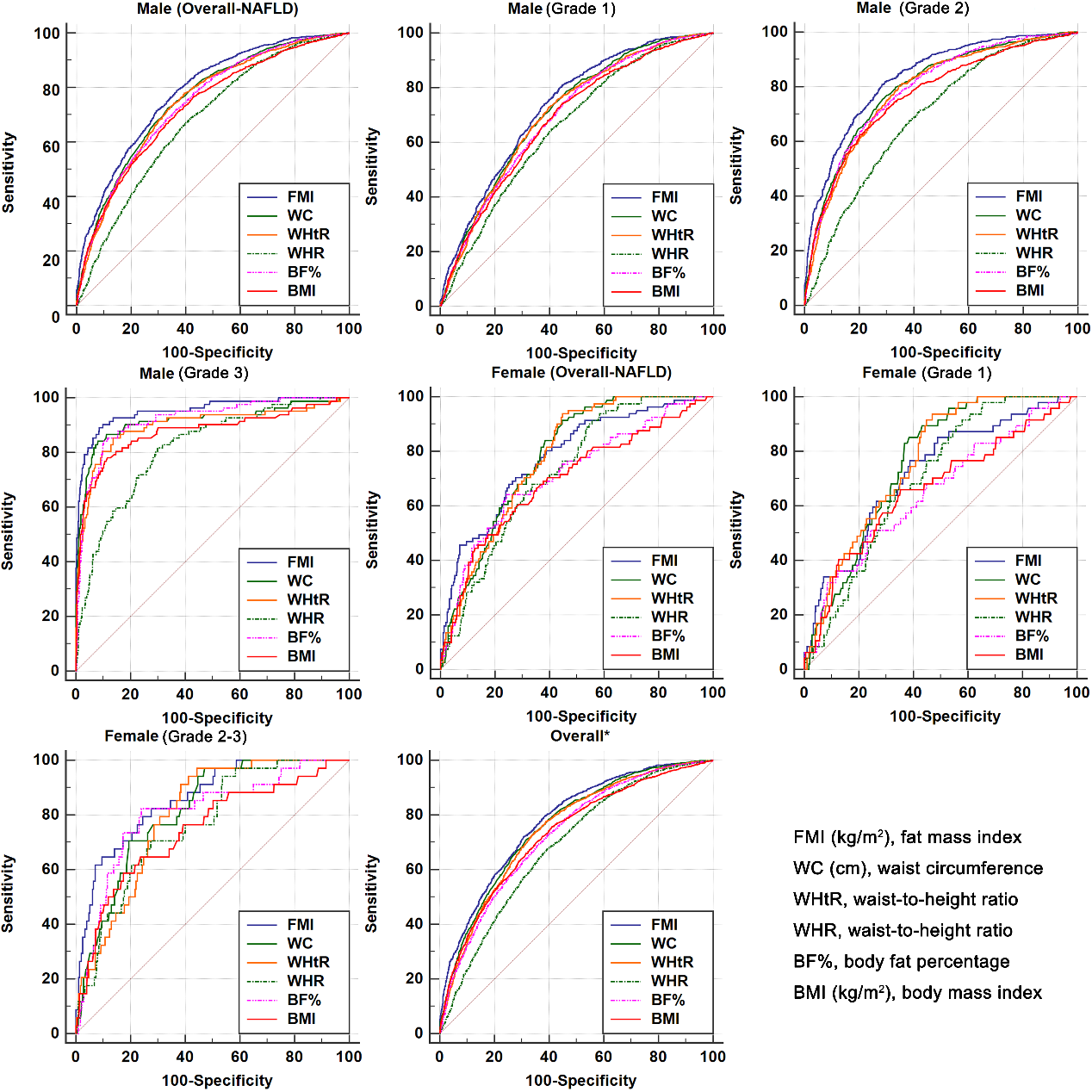


**Fig. S1** ROC curves of different anthropometric indicators for the screening of NAFLD. NAFLD, non-alcoholic fatty liver disease. Overall* indicates the total study population without distinguishing the grade of steatosis. Grade 1 to Grade 3 represents the degree of hepatic steatosis.


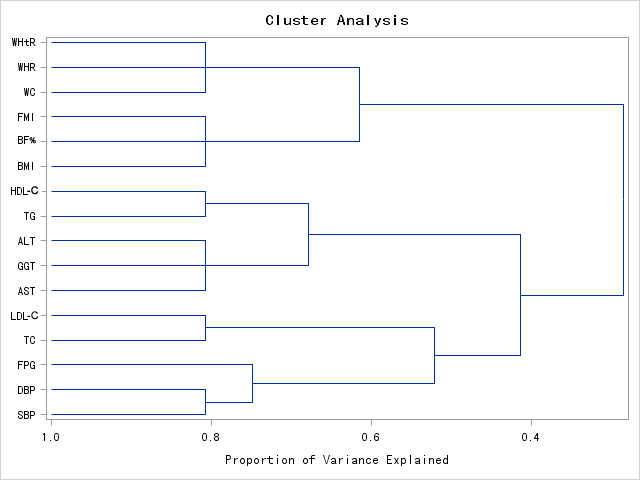


**Fig. S2** Horizontal dendrogram of cluster analysis results. BMI, body mass index; WC, waist circumference; HC, hip circumference; WHR, waist-to-height ratio; WHtR, waist-to-height ratio; BF%, body fat percentage; FMI, fat mass index; SBP, systolic blood pressure; DBP, diastolic blood pressure; FGP, fasting plasma glucose; HDL-C, high-density lipoprotein; LDL-C, low-density lipoprotein; TG, triglycerides; TC, total cholesterol; AST, aminotransferase; ALT, alanine aminotransferase; GGT, γ-glutamyl transferase.


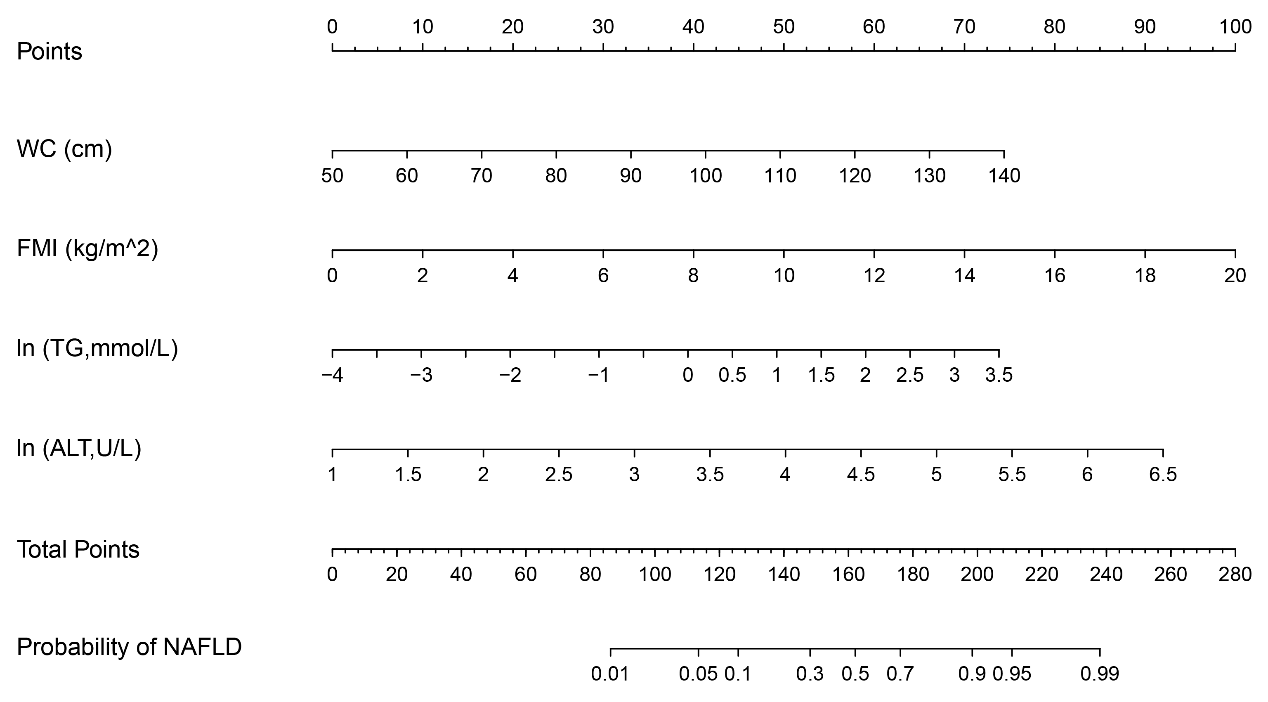


**Fig. S3** The nomogram for NAFLD according to the best subset regression. WC (cm), waist circumference; FMI (kg/m^2^), fat mass index; ln (TG, mmol/L), natural logarithm of serum triglyceride levels; ln (ALT, U/L), natural logarithm of serum alanine aminotransferase levels.


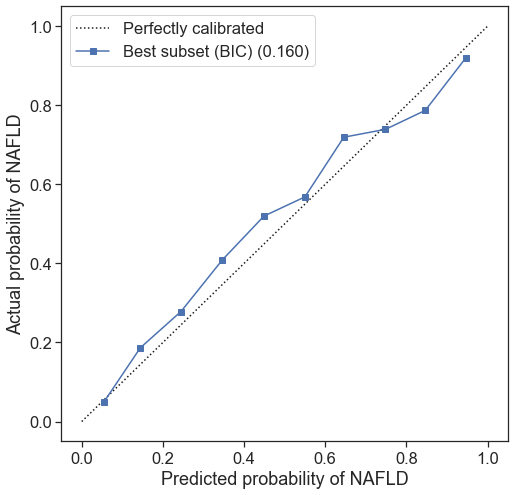


**Fig. S4** The calibration curve for the best subset (BIC) model. BIC, Bayesian information criterion; NAFLD, nonalcoholic fatty liver disease.


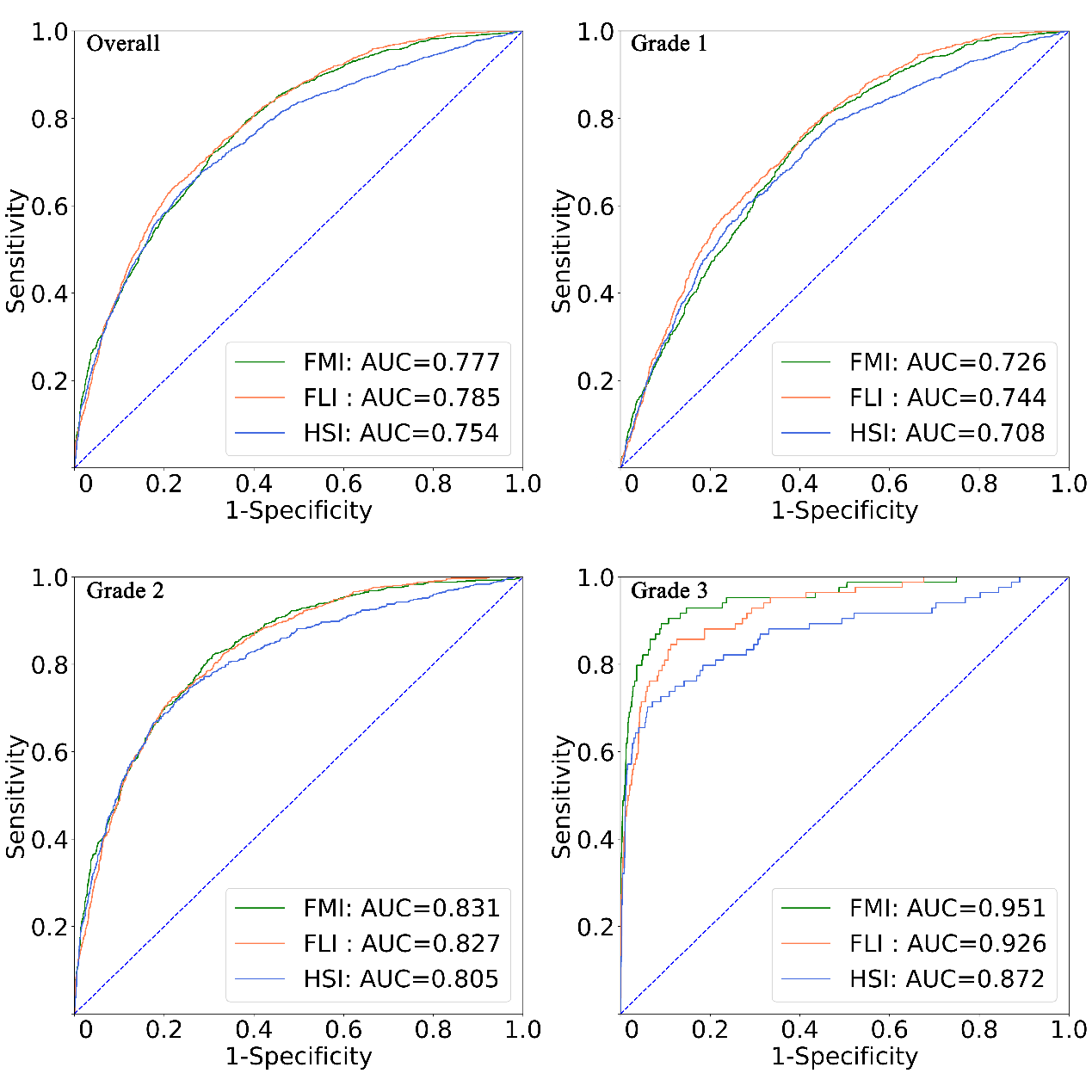


**Fig. S5** ROC curves of different combination panels and FMI for the screening of NAFLD. Overall indicates the total study population without distinguishing the grade of steatosis. Grade 1 to Grade 3 represents the degree of hepatic steatosis.

**References**

1 2016 Chinese guidelines for the management of dyslipidemia in adults. *Journal of geriatric cardiology : JGC* **15**, 1-29, doi:10.11909/j.issn.1671-5411.2018.01.011 (2018).

2 Wong, V. W. *et al.* Validation of the NAFLD fibrosis score in a Chinese population with low prevalence of advanced fibrosis. *The American journal of gastroenterology* **103**, 1682-1688, doi:10.1111/j.1572-0241.2008.01933.x (2008).

3 Gois, P. H. F. & Souza, E. R. M. Pharmacotherapy for hyperuricaemia in hypertensive patients. *Cochrane Database Syst Rev* **9**, Cd008652, doi:10.1002/14651858.CD008652.pub4 (2020).

4 Millwood, I. Y. *et al.* Conventional and genetic evidence on alcohol and vascular disease aetiology: a prospective study of 500 000 men and women in China. *Lancet* **393**, 1831-1842, doi:10.1016/s0140-6736(18)31772-0 (2019).

5 Farrell, G. C., Chitturi, S., Lau, G. K. & Sollano, J. D. Guidelines for the assessment and management of non‐alcoholic fatty liver disease in the Asia–Pacific region: Executive summary. *Journal of Gastroenterology & Hepatology* **22**, 775-777 (2007).
